# TNF-Alpha Inhibitor Prevents Cigarette Smoke Extract-Induced Cell Death in Osteoarthritis-Derived Chondrocytes in Culture

**DOI:** 10.3390/cells14070489

**Published:** 2025-03-25

**Authors:** Débora Levy, Alexandra Fernandes Calllera, Alyne Riani Moreira, Iolanda de Fátima Lopes Calvo Tibério, Pedro Nogueira Giglio, Marco Kawamura Demange, Sergio Paulo Bydlowski, Fernanda Degobbi Tenorio Quirino Dos Santos Lopes

**Affiliations:** 1 Lipids, Oxidation, and Cell Biology Group, Laboratory of Immunology (LIM19), Heart Institute (InCor), Hospital das Clínicas HCFMUSP, Faculdade de Medicina, Universidade de São Paulo, São Paulo 05403-010, Brazil; d.levy@hc.fm.usp.br; 2 Laboratory of Experimental Therapeutics, LIM-20, Department of Clinical Medicine, Hospital das Clínicas HCFMUSP, Faculdade de Medicina, Universidade de São Paulo, São Paulo 01246-903, Brazil; alexandra.callera@gmail.com (A.F.C.); alyne.riani@gmail.com (A.R.M.); iocalvo@uol.com.br (I.d.F.L.C.T.); 3 Thoracic Surgery Research Laboratory (LIM61), Division of Thoracic Surgery, Heart Institute (InCor), Hospital das Clínicas HCFMUSP, Faculdade de Medicina, Universidade de São Paulo, São Paulo 05403-010, Brazil; 4 Institute of Orthopedics and Traumatology, Faculdade de Medicina, Universidade de São Paulo, São Paulo 05403-010, Brazil; pedrongiglio@gmail.com (P.N.G.); marco.demange@hc.fm.usp.br (M.K.D.); 5 National Institute of Science and Technology in Regenerative Medicine (INCT-Regenera), CNPq, Rio de Janeiro 21941-902, Brazil

**Keywords:** chondrocyte, cigarette smoke extract, TNF-α inhibitor, osteoarthritis, apoptosis, caspase 3/7, mitochondrial transmembrane potential, ROS

## Abstract

Smoking has been associated, among other factors, with musculoskeletal disorders. Although there is no consensus about the effects of smoking on osteoarthritis (OA), the increase in TNF-alpha in smokers has been considered an important factor in OA induction or progression. However, studies on the effects of smoking on chondrocytes are lacking. Here we aimed to study the effects of cigarette smoke extract (CSE) associated with a TNF-alpha inhibitor on cell death of primary human chondrocytes derived from osteoarthritic patients. CSE at 10% led to cell death by apoptosis after 48 h of incubation, together with caspase 3/7 activation, decrease in mitochondrial transmembrane potential, ROS production, and improvement in *syndercan-1*, *perlecan*, and *RUNX2* gene expression. All these effects promoted by CSE were reversed by TNF-alpha inhibitor. Collagen II, F-actin, and SOX9 were also analyzed, and CSE promoted alteration in the expression of these proteins. In conclusion, our results support the clinical impact of smoking on OA development by showing the detrimental action of CSE on osteoarthritis-derived chondrocytes and the protective effects of TNF-alpha inhibitors, reinforcing the importance of this cytokine in the cartilage injury process.

## 1. Introduction

Smoking is one of the leading causes of preventable death worldwide and is a major risk factor for lung cancer as well as cardiovascular and respiratory diseases [1]. Cytotoxic components in cigarette smoke composition promote a systemic inflammatory response mediated by pro-inflammatory cytokines, such as tumor necrosis factor alpha (TNF-α), promoting an impairment of different tissue cell activities with a concomitant increase in oxidative stress and apoptosis mechanisms [2,3,4]. The association between smoking and musculoskeletal disorders, such as rheumatoid arthritis, periodontitis, osteoporosis, and fragility fractures, has also been described [5]. Although there is no consensus about the effects of smoking on OA development and progression, some mechanisms have been proposed [6]. Smoking has been shown to impair bone healing by inducing osteoblast apoptosis [7] and increasing osteoclast bone resorption activity [8,9]. In addition, the increase in systemic inflammation observed in smokers could be considered an important factor in OA induction or progression.

Osteoarthritis is a disease characterized by cartilage degradation, and an association with systemic inflammation could also be present. Moreover, chondrocytes, the only cells in cartilage, strictly regulate cartilage architecture and biochemical composition in response to changes in their environmental, chemical, and mechanical stimulus [10]. Under the pro-inflammatory environmental stimulus, they produce several cytokines, including interleukin 1β, interleukin 6, and tumor necrosis factor (TNF) alpha, and matrix-degrading enzymes including the metalloproteinases, which lead to cartilage degeneration [11].

Since TNF-alpha is recognized as a potent pro-apoptotic cytokine [2], different TNF-alpha inhibitors have been used in chronic autoimmune and inflammatory diseases as treatment approaches [12]. It was demonstrated that intraarticular utilization of TNF-alpha inhibitors (adalimumab and infliximab) was able to reduce knee pain in OA patients [13,14,15]. However, the efficiency of these agents in treatment at the cellular level needs better elucidation [16].

The literature on the effects of TNF alpha inhibition in cartilage is highly heterogeneous and comprises a majority of moderate to low-quality studies [12]. Although in vitro cell-based assays are crucial for evaluating the efficacy of new therapeutics in preclinical research, there are several challenges associated with their application. Cell lines often exhibit genetic variations due to inherent mutations or deliberate genetic modifications, which can result in discrepancies in phenotypic traits, functionality, and drug responses when compared to primary cells. Moreover, prolonged subculturing of these cell lines in vitro can lead to genetic instability. In this regard, human osteoarthritic primary chondrocytes (OACs), derived from discarded biomedical materials following joint-replacement surgeries, offer a promising and accessible source of cells for drug testing. Notably, OACs have been shown to maintain genetic stability throughout extended in vitro culture, making them a reliable model for research [16,17,18].

Considering that cigarette smoking induces systemic inflammation and increased TNF-alpha levels and that chondrocyte activity depends on the mechanical and chemical stimulus of the microenvironment, in this study, we aimed to study primary human chondrocytes of osteoarthritic patients and the effects of cigarette smoke extract (CSE) associated with a TNF-alpha inhibitor administration on the cell viability, reactive oxygen species (ROS) expression, mitochondrial activity, extracellular matrix effects, and differentiation gene expression.

## 2. Materials and Methods

### 2.1. Isolation and Culture of Chondrocytes from OA Patients

This study was approved by the Ethics Committee of the Institution (no. 46438821.2.0000.0068 and 1847/2022) and performed in accordance with the Declaration of Helsinki. Informed consent was obtained from three volunteers (1 man and 3 women) undergoing knee replacement treatment due knee osteoarthritis. A 5–10 mm cartilage fragment was removed from a non-weight-bearing area on the lateral edge of the trochlea or intercondylar notch. The samples were washed 3 times in PBS and then mechanically dissociated with a scalpel blade on a 56.5 cm^2^ culture plate (Corning, New York, NY, USA). Homogeneous fragments (≤1 mm) were incubated at 37 °C in a humid atmosphere with 5% CO_2_ for 15 min. Then, 15 mL of Dulbecco’s Modified Eagle’s Medium (DMEM—Sigma-Aldrich, Saint Louis, MO, USA). Low-glucose medium supplemented with penicillin 100 IU/mL, streptomycin 100 µg/mL and 20% (*v*/*v*) fetal bovine serum (Gibco, Karlsruhe, Germany) were added slowly so as to not disturb the cartilage pieces, followed by incubation at 37 °C for 72 h in a humid atmosphere with 5% CO_2_. The medium was then replaced and the non-adherent cartilage fragments were removed. Cells were used for experiments at the 4–6th passage. Control cells were chondrocytes from OA patients cultivated with DMEM medium with 20% SFB and 100 U/mL penicillin and 100 mg/mL streptomycin (basal medium). Data are mean ± SEM from two independent experiments in duplicate for each patient in each tested condition.

### 2.2. Generation of CSE

The 100% CSE was freshly prepared by smoking two commercially cigarettes (0.8 mg of nicotine, 10 mg of tar and 10 mg of CO per cigarette Derby, Souza Cruz, Rio de Janeiro, Brazil) directly into 20.0 mL of DMEM medium with 20% FBS, 100 U/mL penicillin and 100 mg/mL streptomycin utilizing a syringe-based smoking apparatus [19]. The 100% CSE was sterile filtered through a 0.22 µm filter to remove undissolved particulates. The 100% CSE was then normalized to its optical density (OD) at a wavelength of 320 nm [20]. Acceptable OD values for 100% CSE batches were 0.733 ± 0.130.

### 2.3. Assessing Chondrocyte Viability with MTT and PI Staining

Cells were plated at a density of 5 × 10^3^ cells/cm^2^ in 96-well flat-bottom polystyrene microplates (Corning, New York, NY, USA) and incubated for 24 h for cell adherence. After that, cells were incubated with different concentrations of CSE with or without 20 µg/mL of anti-TNF-alpha (adalimumab) for 24 and/or 48 h.

Cell viability was determined using MTT (3-[4,5 dimethylthiazol-2-yl]-2,5 diphenyltetrazolium bromide) [21]. Briefly, 10 µL MTT reagent (Sigma–Aldrich, New York, NY, USA) were added to each well to a final concentration of 5 mg/mL, incubated for 4 h at 37 °C, and then centrifuged at 2000 rpm for 10 min. The medium was discarded, and 100 µL of dimethyl sulfoxide (Sigma–Aldrich, New York, NY, USA) were added to each well. The experiment was performed using six replicates for each concentration and was repeated three times. The amount of formazan was determined by measuring the absorbance at 570 nm refereed to 630 nm using an Elx800™ absorbance microplate reader (Biotek, Winooski, VT, USA). Cell death was proven by a propidium iodide (PI) internalization experiment. After 24 or 48 h of incubation with CSE with or without adalimumab, the cells were incubated with 0.1 μg/mL Hoechst 33342 (Molecular Probes, Eugene, OR, USA) and 0.5 µL propidium iodide (PI) (Molecular Probes, Eugene, OR, USA) for 15 min. After that, the cells were washed with PBS, and the cell death was determined by measuring the fluorescence of PI with excitation at 493 nm and an emission at 636 nm and Hoechst 33342 excitation at 350 nm and an emission at 461 nm using the SpectraMax Paradigm multi-mode detection platform (Molecular Devices, San Jose, CA, USA). Data was analyzed on software Softmax Pro V6.2.1 (Molecular Devices, San Jose, CA, USA).

### 2.4. Caspase 3/7 Activity

The cells were plated at a density of 1.0 × 10^3^ cells/cm^2^ in 96-well flat-bottom polystyrene microplates and treated with CSE at concentrations of 2.5%, 5%, and 10%, with or without adalimumab, for 24 h or 48 h.

Caspase 3/7 activity was measured using CellEvent Caspase-3/7 Green (Invitrogen, Waltham, MA, USA) as described by the manufacturer. The nuclei were counterstained with 0.1 µg/mL Hoechst 33342. Fluorogenic substrates were determined using the SpectraMax Paradigm multi-mode detection platform (Molecular Devices, San Jose, CA, USA). Data was analyzed using the software Softmax Pro V6.2.1 (Molecular Devices, San Jose, CA, USA).

### 2.5. Measurement of Transmembrane Mitochondrial Potential (ΔΨm) 

Cells were treated as previously described. At the end of the experimental period, treated cells were incubated with 50 nM TMRE (Sigma Aldrich, New York, NY, USA) for 30 min at 37 °C. TMRE is a potentiometric and cationic indicator die that accumulates preferentially in energized mitochondria [22]. Nuclei were counterstained with 0.1 µg/mL Hoechst 33342. TMRE fluorescence was determined using the SpectraMax Paradigm multi-mode detection platform (Molecular Devices, San Jose, CA, USA). Data was analyzed using the software Softmax Pro V 6.2.1 (Molecular Devices, San Jose, CA, USA).

### 2.6. Analysis of ROS

ROS were determinate using a DCFDA/H2DCFDA cellular ROS assay kit (Abcam, Cambridge, UK). The chondrocytes were plated at a density of 1.0 × 10^3^ cells/cm^2^ and treated as previously described. At the end of the experimental period, treated cells were incubated with 10 µM DCFH-DA in culture media for 45 min. Then, the cells were washed with buffer provided by the kit and suspended in PBS. Intracellular ROS production was determined using the SpectraMax Paradigm multi-mode detection platform (Molecular Devices, San Jose, CA, USA). Data was analyzed using the software Softmax Pro V. 6.2.1 (Molecular Devices, San Jose, CA, USA).

### 2.7. Protein Detection by Indirect Immunofluorescence

Expression of collagen II, F-actin, and SOX9 was evaluated by indirect immunofluorescence. Cells were seeded into black 96-well flat-bottomed microplates (Corning, New York, NY, USA) at a concentration of 1.5 × 10^3^ cells per well and cultured in complete medium at 37 °C, a humid atmosphere, and 5% CO_2_ for 24 h. After that, the cells were incubated with CSE at concentrations of 2.5%, 5%, and 10%, with or without 20 µg/mL of anti-TNF-alpha (adalimumab), for 48 h.

Then, the cells were washed with 100 μL of Dulbecco’s phosphate-buffered saline (DPBS), followed by the addition of 4% paraformaldehyde (Sigma-Aldrich, Saint Louis, MO, USA) for 2 h at 4 °C. After the incubation period, the plates were washed twice with DPBS and the cells were permeabilized with 0.1% Triton X-100 solution (Sigma-Aldrich, Saint Louis, MO, USA) at 4 °C for 15 min, followed by blocking with BSA 5% (Sigma-Aldrich, Saint Louis, MO, USA) for 40 min at room temperature.

The cells were then incubated for 16 h at 4 °C with collagen II monoclonal antibody (2B1.5—Invitrogen, Walthan, MA, USA) or with SOX9 monoclonal antibody (Ab92494—Abcam, Cambridge, UK). Then, the cells were further incubated for 2 h with secondary anti-rabbit AlexaFluor^®^ 488 antibody (1:500 dilution; Molecular Probes, Eugene, OR, USA). These specific treatments were all followed by 2 h incubation with 0.1 µg/mL Hoechst 33342 dye for nucleus labeling. Then, the cells were analyzed using an Agilent BioTek Cytation C10 confocal imaging reader (Biotek, Winooski, VT, USA). Nine sites per well and two wells per treatment were acquired.

Changes in actin organization were investigated using Alexa Fluor 488 phalloidin (Molecular Probes, San Jose, CA, USA). After a blocking procedure, the cells were incubated with 3 U/mL of phalloidin in DPBS for 30 min. After washing with DPBS, the cell nuclei were stained with 300 nM of DAPI. The plates were then washed twice with DPBS and analyzed using an Agilent BioTek Cytation C10 confocal imaging reader (Biotek, Winooski, VT, USA). Nine sites per well and two wells per treatment were acquired.

### 2.8. Evaluation of Genes Involved in Cell Differentiation

This work is in consonance with the MIQE guidelines regarding minimum information for publication of quantitative real-time PCR experiments [23], as follows: approximately 1.0 × 10^6^ cultured cells in 6-well plates (Corning, New York, NY, USA) with different concentrations of CSE with or without 20 µg/mL of anti-TNF-alpha (adalimumab) for 48 h.

After that, the cells were washed with PBS and 1.0 mL of TRI reagent (Sigma-Aldrich, Saint Louis, MO, USA) was added and incubated for a minimum of 24 h at −20 °C. RNA extraction was performed as described by the manufacturer.

All RNA samples were spectrophotometrically quantified (NanoDrop 1000 Spectrophotometer V3.7, Thermo Fisher Scientific, Waltham, MA, USA). Before the synthesis of cDNA, the RNA (1 µg) was incubated with RQ1 RNase-free DNAse (Promega, Madison, WI, USA). The cDNA synthesis was performed using a high-capacity cDNA reverse transcription kit (Applied Biosystems, Waltham, MA, USA). The incubation was performed using a thermal cycler (XP Cycler, Bioer technology, Hangzhou, China).

The analysis of expression of genes related to osteogenic (RUNX2), adipogenic (PPARγ), and chondrogenic (syndecan and perlecan) cell differentiation was performed by real-time PCR. The mRNA expression was normalized based on the expression of the endogenous GAPDH gene (Thermo Fisher, Waltham, MA, USA). The assays were performed in duplicate, and the variation in cycle of threshold (CT) values between the duplicates did not exceed 0.5. Each assay included a reference sample and a negative amplification control, without adding a sample. A 7500 Fast Real-Time PCR System thermocycler (Life Technologies, Carlsbad, CA, USA) was used with the following conditions: 95 °C for 5 min, followed by 40 cycles of 95 °C for 15 s and 60 °C for 30 s. The primers were obtained from Integrated DNA Technologies (IDT, Coraville, IA, USA ) using pre-designed assays for hydrolyzable probes (RUNX2—NM_001024630(3); PPARγ—NM_005037(4); Syndercan-1—NM_002997; Perlecan—NM_005529).

### 2.9. Statistics

For IC_50_ calculations, survival data were evaluated by variable slope curve-fitting. For group comparation, ANOVA and Tukey’s post-hoc test were performed using the statistical package GraphPad Prism V.9.0 (GradPad Software, Los Angeles, CA, USA). The results were expressed as mean ± SD of two different experiments carried out in duplicate, considering a statistically significant *p* value < 0.05.

## 3. Results

### 3.1. Effects of CSE on Chondrocyte Viability and Death

**Figure 1:**
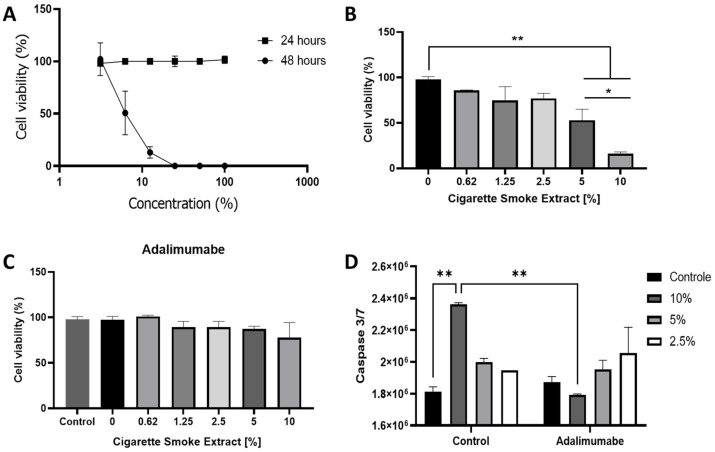
Effects of CSE with or without adalimumab in cell death. (**A**) Cytotoxicity of CSE incubation for 24 and 48 h; (**B**) effect of CSE on chondrocytes treated with different concentrations of CSE (0 to 10%) after 48 h; (**C**) effect of CSE on chondrocytes treated with different concentrations of CSE (0 to 10%) and adalimumab (20 µg/mL) after 48 h; (**D**) caspase 3/7 activity. Ratio of the fluorescence intensity of caspase 3/7 and Hoechst of chondrocytes treated with different concentrations of CSE (0 to 10%) in the presence or absence of adalimumab (20 µg/mL). Analysis of caspase 3/7 was performed by fluorimetry (emission: 502 nm; excitation: 530 nm) and Hoechst 33342 (emission: 461 nm; excitation: 360 nm). Control cells were chondrocytes from OA patients cultivated in basal medium. Data are mean ± SEM from two independent experiments in duplicate. * *p* < 0.05; ** *p* < 0.01.

A concentration- and time- dependent curve was carried out using MTT. CSE had no effect on cell viability and death after 24 h of incubation in any concentration tested. After 48 h, 100% cell death was observed with CSE concentrations higher than 25%. No cell death was observed at concentrations of 0.62%; 1.25%, and 2.5%. The IC_50_ was 6.49 ± 1.5% (Figure 1A,B). To investigate the capacity of TNF-alpha inhibitor in preventing cell death promoted by CSE, 20 µg/mL adalimumab was tested with and without CSE. Adalimumab alone did not affect the chondrocytes (Figure 1C control vs. 0) and was able to decrease cell death promoted by the CSE (Figure 1C) in any tested concentration. The effects of adalimumab on cell death were confirmed by an iodide propidium test (Supplementary Figure S1).

Caspase 3/7 activity was measured to evaluate apoptosis. Cells treated with CSE 5% and 10% had increased activity of caspase 3/7 (Figure 1D). No activity of caspase 3/7 was observed in the presence of adalimumab with or without CSE.

CSE promoted cell death after 48 h in concentrations higher than 5%, at least in part by apoptosis, as demonstrated by the activity of caspase 3/7. Adalimumab was able to prevent cell death promoted by CSE.

### 3.2. Mitochondrial Transmembrane Potential (ΔΨm) Changes and ROS Production Promoted by CSE

**Figure 2:**
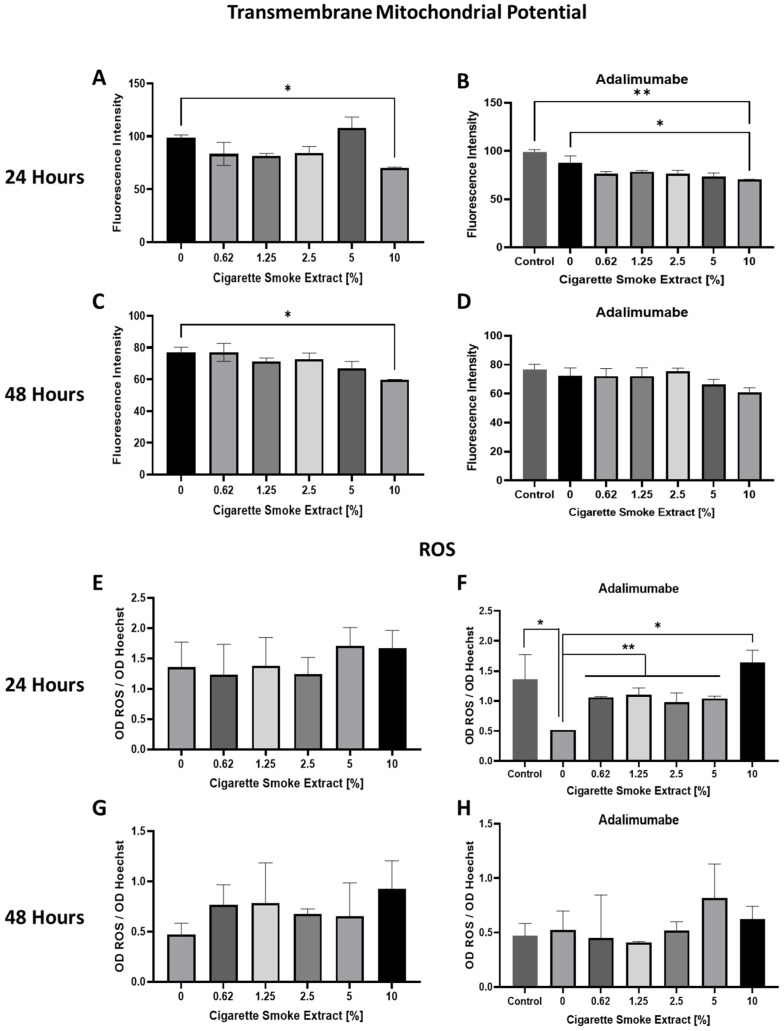
Effects of CSE with or without adalimumab in mitochondrial transmembrane potential and ROS production. (**A**) Mitochondrial ΔΨ of chondrocytes treated with different concentrations of CSE (0 to 10%) after 24 h. (**B**) Mitochondrial ΔΨ of chondrocytes treated with different concentrations of CSE (0 to 10%), in the presence of adalimumab (20 µg/mL) after 24 h. (**C**) Mitochondrial ΔΨ of chondrocytes treated with different concentrations of CSE (0 to 10%) after 48 h. (**D**) Mitochondrial ΔΨ of chondrocytes treated with different concentrations of CSE (0 to 10%) in the presence of adalimumab (20 µg/mL) after 48 h. (**E**) ROS production analysis of chondrocytes treated with different concentrations of CSE (0 to 10%) after 24 h. (**F**) ROS production analysis of chondrocytes treated with different concentrations of CSE (0 to 10%) in the presence of adalimumab (20 µg/mL) after 48 h. (**G**) ROS production analysis of chondrocytes treated with different concentrations of CSE (0 to 10%) after 48 h. (**H**) ROS production analysis of chondrocytes treated with different concentrations of CSE (0 to 10%) in the presence of adalimumab (20 µg/mL) after 48 h. Mitochondrial ΔΨ was evaluated by TMRE performed by fluorimetry (emission: 549 nm; excitation: 574 nm) ROS production analyzed by CellEvent Caspase-3/7 Green (emission: 485 nm; excitation: 535 nm) and Hoechst 33342 (emission: 461 nm; excitation: 360 nm). Controls cells were chondrocytes from OA patients cultivated in basal medium. Data are mean ± SEM from two independent experiments in duplicate. * *p* < 0.05; ** *p* < 0.01.

Mitochondrial ΔΨ was measured through TMRE fluorescence intensity analysis in chondrocytes in the presence of CSE at several concentrations after 24 and 48 h incubation. 10% CSE decreased ΔΨm after 24 and 48 h (Figure 2A,C). Adalimumab 20 µg/mL did not change ΔΨm (Figure 2B,D). After 24 h, the use of 10% CSE with adalimumab decreased ΔΨm (Figure 2B). However, after 48 h, the presence of adalimumab inhibited the effects of 10% of CSE (Figure 2D).

The effect of CSE on ROS production was also evaluated after 24 and 48 h incubation. There was no difference in ROS production in any concentration of CSE tested (Figure 2E,G). Adalimumab alone decreased ROS production after 24 h of incubation (Figure 2F), which returned to control levels after 48 h (Figure 2H). Cells treated with adalimumab and different concentrations of CSE showed an increase in ROS production when compared to cells treated with adalimumab alone (Figure 2F). However, ROS production did not change when cells treated with CSE and adalimumab were compared to nontreated cells (Figure 2F,H) after 24 and 48 h.

CSE decreased ΔΨm at 10% concentration in 24 and 48 h. Adalimumab was able to restore mitochondrial changes after 48 h. ROS production in control cells was inhibited by adalimumab only at 24 h. The use of CSE did not promote changes in ROS production in 24 and 48 h.

### 3.3. Effects of CSE on Collagen II, SOX9, and F-Actin

**Figure 3:**
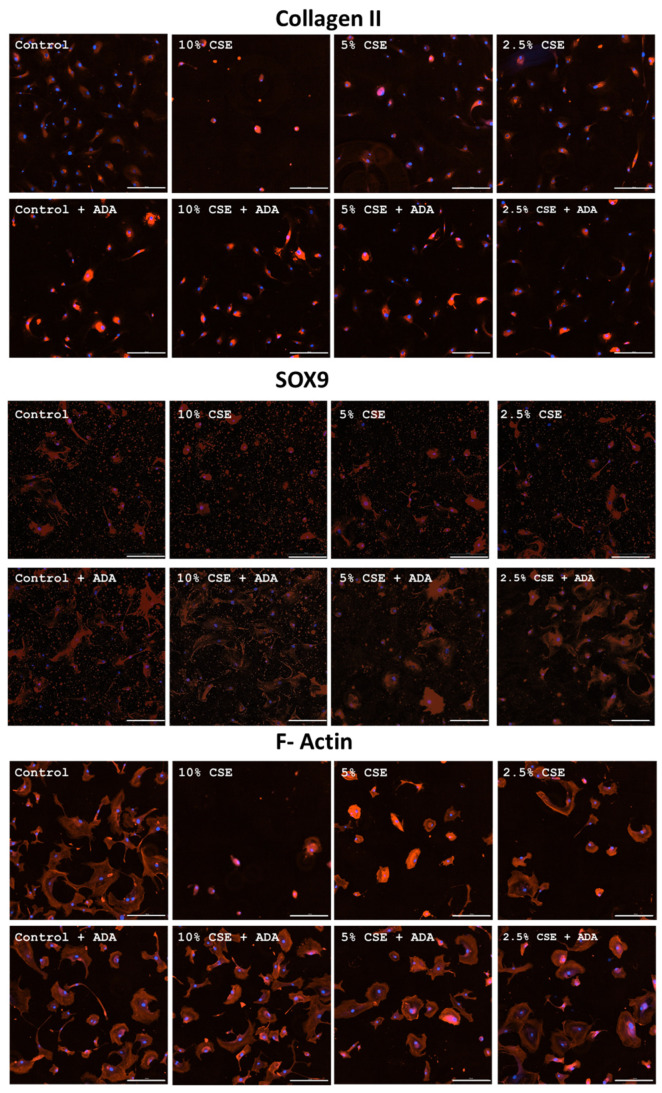
Collagen II, SOX9, and F-Actin expression. Representative figures of cells cultivated with different concentrations of cigarette smoke extract with or without adalimumab (ADA). Expressions measured by immunofluorescence in chondrocytes cultured for 48 h. Scale bar, 100 µM.

Control chondrocytes exhibited a basal level of expression of type II collagen after 48 h of incubation. Incubation of cells with 10% CSE led to a decrease in the number of cells because of cell death; cells had a smaller cytoplasm area and increased fluorescence intensity. When cells were incubated with 5% and 2.5% CSE, there was an increase in the amount of type II collagen without changes in the size of the cytoplasm area when compared to control cells (Figure 3). Cells incubated with adalimumab alone had an increased amount of type II collagen. When the cells were incubated with 10.5 and 2.5% of CSE and adalimumab, the expression of collagen II was the same as in the cells incubated only with adalimumab concentrations that were higher than that of the control cells. CSE did not interfere with this higher expression.

Fluorescence analysis showed that control cells have basal SOX9 expression. When cells were incubated with 10% CSE for 48 h, there was a decrease in the number of cells per field. These cells had a smaller cytoplasmic area. When cells were incubated with 5% and 2.5% CSE, no difference was noted in comparison with control cells (Figure 3).

Cells incubated for 48 h with adalimumab alone showed an increase in cytoplasmic area, with a higher amount of SOX9 than the control without adalimumab. Incubation with 10% CSE and adalimumab did not decrease the number of cells in relation to 10% CSE without adalimumab, but the cell area was increased, similar to cells incubated with adalimumab. When the cells were incubated with 5% and 2.5% CSE and adalimumab, it was observed that the expression of SOX9 was the same as in the cells incubated with adalimumab alone (Figure 3).

Control cells have basal F-actin expression after 48 h incubation. When cells were incubated with 10% cigarette extract, there was a decrease in the number of cells per field, and these cells had a smaller area of cytoplasm. When cells were incubated with 5% cigarette extract, an increase in the amount of cytoplasmic F-actin and a decrease in the area of cytoplasm were observed. Incubation with 2.5% cigarette extract did not lead to any change in the amount of F-actin, but still, the presence of a smaller area of the cultured chondrocytes was observed (Figure 3). Cells incubated only with adalimumab or with 10%, 5%, and 2.5% CSE and adalimumab did not present any change in relation to control cells without adalimumab.

CSE interfered with the expression of collagen II and SOX9 in the tested concentrations. Adalimumab alone is able to increase expression of collagen II and SOX9 independent of the presence or absence of CSE in these proteins. F-actin protein is affected by CSE, and adalimumab was able to revert its effects.

### 3.4. Analysis of Perlecan and Syndercan-1 Gene Expression

**Figure 4:**
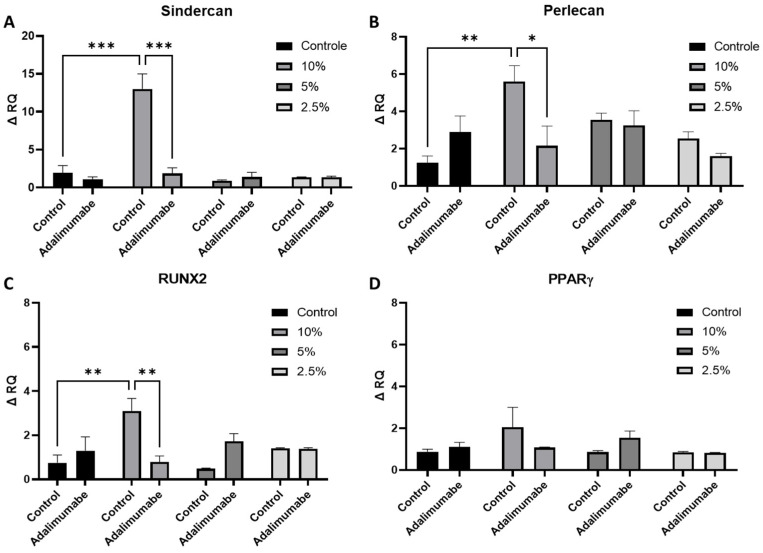
*Syndercan-1*, *perlecan*, *RUNX2*, and *PPARγ* gene expression in chondrocytes treated with different concentrations of CSE (0% to 10%), in the presence or absence of adalimumab (20 µg/mL). (**A**) *Syndercan-1*. (**B**) *Perlecan*. (**C**) *RUNX2*. (**D**) *PPARγ*. ANOVA and Tukey’s post-hoc test were performed to compare groups. RQ (relative quantification) data are presented as mean and standard error (SEM). Analyses performed using GraphPad Prism Prism V. 9 software. Controls cells were chondrocytes from OA patients cultivated in basal medium. * *p* < 0.05; ** *p* < 0.01; *** *p* < 0.001.

The chondrocytes used in the experiments come from human cartilage and were obtained through cell migration. They were capable of differentiating into osteogenic, adipogenic, and chondrogenic lineages. Therefore, we also analyzed genes involved in these differentiations and regulation of cartilage. The *perlecan* and *syndercan-1* genes are involved in regulating cartilage homeostasis. When chondrocytes were treated with 10% CSE, an increase in the expression of both the *perlecan* and *syndercan-1* genes were observed (Figure 4A,B). The use of adalimumab reversed the effect caused by CSE.

### 3.5. Analysis of RUNX2 and PPARγ Gene Expression

The *RUNX2* gene, in addition to being involved in the final phases of chondrogenic maturation, is also involved in osteogenic differentiation. When chondrocytes were treated with 10% CSE, an increase in the expression of the *RUNX2* gene was observed. The use of adalimumab impaired the effect promoted by CSE. Adalimumab did not lead to any change in *RUNX2* gene expression, nor did the concentrations of 5% and 2.5% of CSE (Figure 4C). No alterations were found in the *PPARγ* gene in any of the conditions tested (Figure 4D).

## 4. Discussion

**Figure 5:**
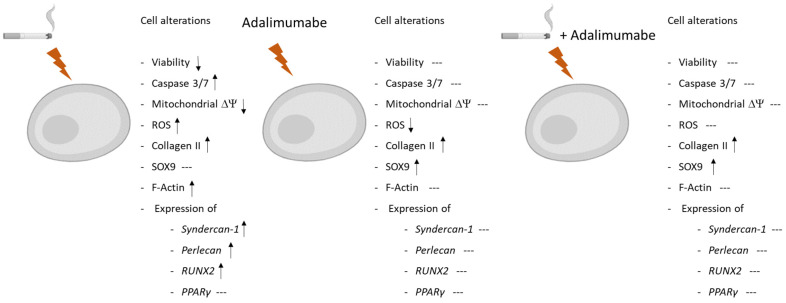
Effect of CSE and CSE plus adalimumab on human chondrocyte culture. The deleterious effect of CSE on viability was demonstrated by caspase 3/7, mitochondrial ΔΨ, and ROS production. Also demonstrated were alterations in collagen II, F-actin, and in expression of *syndercan-1*, *perlecan*, and *RUNX2*. Adalimumab itself promotes decreased ROS production and an increase in collagen II and SOX9. CSE and adalimumab, used together, prevent the deleterious effects of CSE. ↑: Increase; ↓: decrease and ---: no effect.

Here we described the effects of CSE on osteoarthritis-derived chondrocytes, showing the alteration of mitochondrial activity, which, together with the increase in ROS expression, has culminated in the apoptosis of chondrocytes. Furthermore, it has been shown that the administration of TNF-alpha inhibitors reverted these effects, attesting to the fundamental role of TNF-alpha in these events (Figure 5).

The cytotoxic effect of CSE was tested by MTT and PI staining methods for detecting cell death. No effect was observed after 24 h; after 48 h, the CSE IC50 was 6.49%. The intrinsic apoptosis signaling pathway is triggered by stimuli that generate intracellular signals targeting specific components within the cell [24]. This process initiates events at the mitochondria, resulting in mitochondrial dysfunction, followed by the release of cytochrome c and the activation of caspases 9 and 3 [25]. We found caspase 3/7 activation and mitochondrial dysfunction, corroborating cell death by intrinsic apoptosis. Adalimumab was able to reverse all the effects caused by cigarette exposure that led to cell death, preventing the apoptosis stimuli.

The inflammatory response induced by cigarette smoking has been recognized as playing a pivotal role in the occurrence of oxidative stress and apoptosis metabolism, and it has been described mainly in respiratory diseases such as chronic obstructive pulmonary disease (COPD) [26]. However, Junqueira et al. [27] showed, in a CS-induced model of COPD, not only structural changes in the lungs, but also in the bones, and the TNF-alpha increase was associated with these events.

The systemic inflammatory response in smokers, mediated by innate and adaptative immune cells [28], has been considered an important factor in the development of OA [29], but it is not a consensus. Considering that most studies are based on radiographic images and their associations with clinical symptoms, there is a lack of investigation on how chondrocyte metabolism under CS exposure could contribute to this disease development.

In this context, in this study, we evaluated the effects of CSE on cell markers, which could improve the understanding of chondrocyte differentiation and adhesion and how it could influence collagen type II production.

The analysis of SOX9 expression has shown that the administration of cigarette extract lower than 5% did not cause any alteration of this marker in chondrocytes. However, the administration of adalimumab alone induced an overexpression of this marker in these cells. SOX-9 is a protein involved in chondrocyte differentiation, and its overexpression may be associated with an increase in the density of these cells in the cartilage tissue [30].

The SOX9 data are consistent with those observed for type II collagen expression. The adalimumab administration alone also promoted an increase in type II collagen expression in the chondrocyte cultures. This increased differentiation of these cells is possibly associated with the increased production of this matrix protein.

Regarding type II collagen expression, the administration of cigarette extract at a 10% concentration caused alterations in this matrix component. However, the administration of the TNF-alpha inhibitor prevented this alteration.

We also evaluated F-actin, a protein that constitutes the cellular cytoskeleton, and observed that the 10% cigarette extract also altered this protein. However, treatment with the TNF-alpha inhibitor avoided these effects. Cellular cytoskeleton proteins are important for cell adhesion to tissues, among other functions. In the case of chondrocytes, they are crucial for their adhesion to cartilage and, consequently, for type II collagen production [31].

The present study analyzed the expression of factors related to the cell differentiation genes *RUNX2, syndecan-1, perlecan,* and *PPARγ*. *RUNX2*, a transcription regulator for type X collagen and an established marker for chondrocyte hypertrophy, is involved in the calcification and degradation of cartilage matrices and directly implicated in the pathogenesis of osteoarthritis [32]. Our results indicate that CSE at 10% significantly increased *RUNX2* gene expression, and the use of TNF-alpha inhibitor can restore it to basal conditions. Heparan sulfate (HS) proteoglycans bind to many proteins that regulate cartilage homeostasis, including growth factors, morphogens, proteases, and their inhibitors, and modulate their localization, retention, and biological activity [33]. It is a critical regulator of chondrocyte homeostasis and cartilage health [34]. The major HS proteoglycan in cartilage is *perlecan*, and another important HS proteoglycan is *syndecan-1*. Previous studies have shown increased expression of both proteins in OA patients [35,36]. Both genes were overexpressed with treatment of CSE at 10% and this was avoided with treatment with TNF-alpha inhibitor. Peroxisome *proliferator-activated receptor gamma* (*PPARγ*) is a ligand-induced transcription factor known to be involved in normal cell function. Previous research has shown that *PPARγ* is a key regulator of cartilage health [37]. Existing evidence demonstrated that activation of *PPARγ* could alleviate apoptosis and protect chondrocytes [38]. Our results demonstrated that CSE as well as TNF-alpha inhibitor did not change *PPARγ* gene expression.

## 5. Conclusions

This study demonstrates the effects of CSE on the worsening of osteoarthritis chondrocytes through the increase in TNF-alpha, reinforcing the importance of this cytokine in cartilage injury and its role as a therapeutic target in joint diseases. Additionally, these results provide insights into the detrimental effects of CSE on chondrocytes, supporting previous clinical findings that have demonstrated the impact of smoking on OA development.

## Data Availability

The data generated in this research can be made available upon consultation with SPB or FDTQDS. The data will be made available anonymously. Data from medical records of study participants cannot be made available.

## Supplementary Materials

The following supporting information can be downloaded at: https://www.mdpi.com/article/10.3390/cells14070489/s1, Figure S1: Effects of CSE, with or without adalimumab, on cell death. A: Effect of CSE in chondrocyte cells treated with different concentrations of CSE (0 to 10%) after 48 h. B: Effect of CSE in chondrocyte cells treated with different concentrations of CSE (0 to 10%) and adalimumab (20 µg/mL) after 48 h. Analysis performed by fluorimetry of PI (emission: 493 nm; excitation: 636 nm) and Hoechst 33342 (emission: 461 nm; excitation: 360 nm).

## Abbreviations

The following abbreviations are used in this manuscript:

AO	osteoarthritis
TNF-alpha	tumor necrosis factor-alpha
CSE	cigarette smoke extract
ROS	reactive oxygen species
SEM	standard error of the mean
FBS	fetal bovine serum
OD	optical density
MTT	3-[4,5 dimethylthiazol-2-yl]-2,5 diphenyltetrazolium bromide
PI	propidium iodide
CT	cycle threshold
ΔΨm	mitochondrial transmembrane potential
COPD	chronic obstructive pulmonary disease
CS	cigarette smoke
HS	heparan sulfate
PPARγ	peroxisome proliferator-activated receptor gamma
