# Supplementary material for: TNF-Alpha Inhibitor Prevents Cigarette Smoke Extract-Induced Cell Death in Osteoarthritis-Derived Chondrocytes in Culture
Source: Cells. 2025 Mar 25;14(7):489. doi: 10.3390/cells14070489 (PMC11988143; doi:10.3390/cells14070489)
Supplement: Supplementary file 1 [file cells-14-00489-s001.zip › cells-3490665-supplementary.pdf]

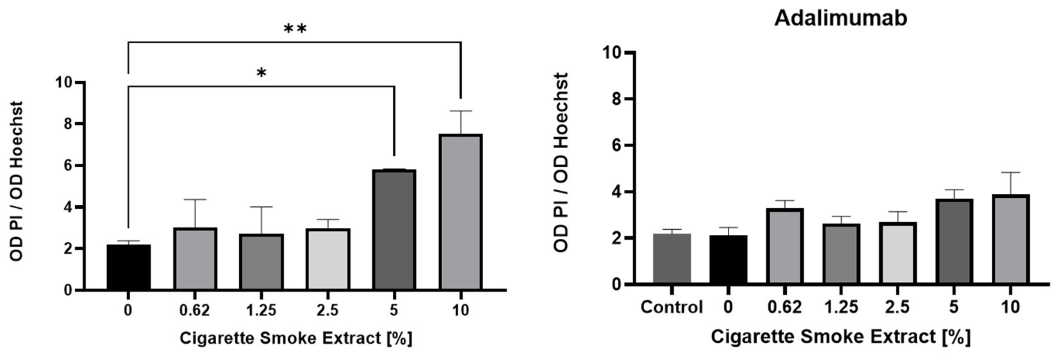

Figure S1: Effects of CSE with or without adalimumab in cell death. A: Effect of CSE in chondrocytes cell treated with different concentrations of CSE (0 to 10%), after 48 hours. B: Effect of CSE in chondrocytes cell treated with different concentrations of CSE (0 to 10 %) and adalimumab (20 µg/mL), after 48 hours. Analysis performed by fluorimetry of PI (emission: 493 nm; excitation: 636nm) and Hoechst 33342 (emission: 461 nm; excitation: 360 nm). Control cells were chondrocytes from OA patients cultivated in basal medium. Data are mean ± SEM from two independent experiments in duplicate. * $p < 0.05$; ** $p < 0.01$.
